# Associations between increased circulating endothelial progenitor cell levels and anxiety/depressive severity, cognitive deficit and function disability among patients with major depressive disorder

**DOI:** 10.1038/s41598-021-97853-9

**Published:** 2021-09-14

**Authors:** Ying-Jay Liou, Mu-Hong Chen, Ju-Wei Hsu, Kai-Lin Huang, Po-Hsun Huang, Ya-Mei Bai

**Affiliations:** 1 Department of Psychiatry, Taipei Veterans General Hospital, No. 201, Shih-Pai Road, Sec. 2, 11217 Taipei, Taiwan; 2 Department of Psychiatry, School of Medicine, National Yang Ming Chiao Tung University, Taipei, Taiwan; 3 Institute of Brain Science, National Yang Ming Chiao Tung University, Taipei, Taiwan; 4 Division of Cardiology, Department of Medicine, Taipei Veterans General Hospital, No. 201, Shih-Pai Road, Sec. 2, 11217 Taipei, Taiwan; 5 Department of Critical Care Medicine, Taipei Veterans General Hospital, Taipei, Taiwan; 6 Cardiovascular Research Center, Taipei Veterans General Hospital, Taipei, Taiwan

**Keywords:** Biomarkers, Diseases, Medical research

## Abstract

The association of major depressive disorder (MDD) with cardiovascular diseases (CVDs) through endothelial dysfunction is bidirectional. Circulating endothelial progenitor cells (cEPCs), essential for endothelial repair and function, are associated with risks of various CVDs. Here, the relationship of cEPC counts with MDD and the related clinical presentations were investigated in 50 patients with MDD and 46 healthy controls. In patients with MDD, a battery of clinical domains was analysed: depressed mood with Hamilton Depression Rating Scale (HAMD) and Montgomery–Åsberg Depression Rating Scale (MADRS), anxiety with Hamilton Anxiety Rating Scale (HAMA), cognitive dysfunction and deficit with Digit Symbol Substitution Test (DSST) and Perceived Deficits Questionnaire-Depression (PDQ-D), somatic symptoms with Depressive and Somatic Symptom Scale (DSSS), quality of life with 12-Item Short Form Health Survey (SF-12) and functional disability with Sheehan Disability Scale (SDS). Immature and mature cEPC counts were measured through flow cytometry. Increased mature and immature cEPC counts were significantly associated with higher anxiety after controlling the confounding effect of systolic blood pressure, and potentially associated with more severe depressive symptoms, worse cognitive performance and increased cognitive deficit, higher social disability, and worse mental health outcomes. Thus, cEPCs might have pleiotropic effects on MDD-associated symptoms and psychosocial outcomes.

## Introduction

Major depressive disorder (MDD) is the third leading cause of years lived with disability in 2007 and 2017^1^ and has been projected as the second-leading cause of disability-adjusted life years (DALYs) by 2030^2^. Following MDD, ischaemic heart disease and cerebrovascular disease have been projected respectively as the third and sixth leading causes of DALYs by 2030^2^. Considerable evidence indicates that depression increases cardiovascular disease (CVD) risk. For instance, among individuals who were free of medical illness at baseline, MDD was associated with a more than fourfold risk of myocardial infarction (MI) after control of both other psychiatric diagnoses and medical risk factors^3^. Patients with MDD also had an increased risk of coronary artery disease (CAD)^3^. In addition to categorical MDD diagnosis, the risk of CAD is proportional to the severity of depressed mood^4^. By contrast, patients with CVD are more likely to have depression than the general population. For instance, MDD prevalence is threefold higher in patients with CVD than in the general population^5,6^. More than half of patients with unstable angina, MI or CAD have clinically significant depressive symptoms^6^, and depression is an independent risk factor of a worse outcome after a cardiovascular event^7^. MDD may also increase the risks of atherosclerosis, hypertension, and stroke, even suggesting mutual causality among these conditions^3,8^. This evidence suggests bidirectional and reciprocal relationships between MDD and CVDs^9^.

Several pathophysiological mechanisms underlying the bidirectional association of MDD and CVDs have been proposed, such as unhealthy lifestyles or behaviours (diet, smoking, physical inactivity)^10–12^, pleiotropic effects of shared genetic factors and epigenetic modulations and alterations in gene expression^9^, stress and hypothalamic–pituitary–adrenocortical axis dysfunction^13,14^, inflammation^15–17^, increased platelet reactivity^13^, and endothelial dysfunction attributed to the count and functions of circulating endothelial progenitor cells (cEPCs)^9,18^.

cEPCs, first isolated from human peripheral blood by Asahara et al.^19^, express numerous haematopoietic stem cell-specific (e.g., CD34 and CD133), progenitor cell-specific (e.g., CD133) and endothelial cell-specific [e.g., kinase insert domain receptor (KDR)] surface markers. cEPCs may be divided into immature (or early) and mature (or late) cEPCs according to the presence of particular surface markers: immature cEPCs are positive for CD34, CD133, and KDR, whereas mature cEPCs are positive for CD34 and KDR^20,21^. In response to hypoxia after MI and in microvascular ischaemia, cEPCs are differentiated from bone marrow–derived haematopoietic stem cells^22^, migrate towards sites of vascular injury, incorporate into the endothelial monolayer, and subsequently promote vascular repair and angiogenesis via paracrine signalling to neighbouring cells and transdifferentiating to mature endothelial cells^23,24^. cEPC count is correlated with flow-mediated brachial artery reactivity and carotid intima media thickness, the clinical surrogates of endothelial dysfunction^25,26^, as well as associated with the combined Framingham risk factor score^26^.

A considerable amount of evidence indicating that cEPC count is associated with various CVDs, such as CAD, MI, and ischaemia^27^. Moreover, a decreased cEPC count can increase hazard ratios for cardiovascular events, cardiovascular death, or all-cause death^18^, and a decreased count and functional impairment (reduced mobilisation) of cEPCs are significantly correlated with hypertension—a relationship strengthened by the effects of antihypertensive drugs on the mechanisms of action of cEPCs^28^. In cerebrovascular diseases (another category of major CVDs), increased cEPC counts have been associated with reduced infarct growth and intracerebral haemorrhage volume as well as positive neurological and functional outcomes^29–31^. These findings indicate the pivotal role and intervention potential of cEPCs on the development, course, and associated outcome of CVDs.

Because endothelial dysfunction might be a mechanism underlying the bidirectional MDD–CVD relationship and accumulated evidence suggests cEPCs’ involvement in endothelial dysfunction and CVDs, several studies have investigated the cEPC–depressed mood and cEPC–MDD relationship in patients with or without CVDs and with or without psychosocial stressors^32–41^. These findings imply a potential role of cEPCs in moderating MDD risk and depressive symptoms and need to be replicated in other independent populations. Furthermore, because patients with MDD typically have comorbid cognitive dysfunction^42^, somatic symptoms^43,44^, and impaired quality of life^45^, studying the relationship of cEPCs with the aforementioned clinical parameters during the MDD course is essential. Therefore, the present study investigated whether the cEPC count are associated with MDD and correlated with other clinical presentations, including cognitive dysfunction, somatic symptoms, subjective disability in important functional domains, and quality of life.

## Results

### cEPC counts in MDD patients and HCs

The study enrolled 50 patients with MDD and 46 HCs. The MDD patients were treated with the following antidepressants: Sertraline (n = 10), Escitalopram (n = 7), Fluoxetine (n = 1), Paroxetine (n = 1), Duloxetine (n = 1), Venlafaxine (n = 2), Bupropion (n = 7), Mirtazapine (n = 3) and Bupropion (n = 7). There were two patents treated with two antidepressants simultaneously. Table 1 lists the demographic, clinical, and laboratory data and cEPC counts in the MDD and HC groups. Continuous variables were presented as the medians and interquartile ranges (IQR). The between-group differences in gender distribution or median age, height, body weight, or body mass index were all nonsignificant. The medians of biochemical parameters, such as ALT, CREAT, FBS, UA, FBS, CHOL, TG, or HDL-C levels, also did not differ significantly between the two groups. However, the medians of systolic and diastolic blood pressures (SBP and DBP) were higher in patients with MDD than in HCs, while the median of AST in MDD was significantly lower than that in HCs (Table 1, Mann–Whitney *U* test, all *p* < 0.05). Unfortunately, although the medians of mature and immature cEPC counts in patients with MDD were numerically higher than that in HCs, both cEPC counts were not statistically different in the two groups (Table 1, Mann–Whitney *U* test, all *p* > 0.05).

**Table 1:** Comparison of demographic parameters, clinical measurements, and cEPC counts between patients with major depressive disorder (MDD) and healthy controls (HCs).

	MDD (n = 50)	NC (n = 46)	p value
Gender (male/female)	17/33	17/29	0.092
Age	26 (20)	28 (12.25)	0.716
Illness duration (years)	2 (5.925)	–	
Treatment duration (years)	0.2 (0.515)	–	
Height (cm)	165.25 (15.25)	162 (14.25)	0.323
BW (kg)	63.45 (16.725)	60 (15)	0.348
BMI	22.8616 (5.8062)	22.4294 (3.504)	0.626
HipC (cm)	96 (9)	93 (12)	0.395
WC (cm)	79.5 (16.25)	77 (15)	0.920
SBP (mmHg)	114 (20.75)	111.5 (15)	***0.029***
DBP (mmHg)	70.5 (13.25)	65.5 (11.25)	***0.002***
Heart rate	76 (13)	72.5 (11.25)	0.162
ALT (U/L)	14 (10.25)	15 (9)	0.359
AST (U/L)	16 (5.75)	19 (6.5)	***0.023***
CREAT (mg/dL)	0.76 (0.13)	0.73 (0.15)	0.230
UA (mg/dL)	5.2 (1.75)	5.6 (1.55)	0.360
FBS (mg/dL)	81 (18)	87 (10)	0.098
CHOL (mg/dL)	182 (39)	192 (56)	0.473
TG (mg/dL)	84 (68.75)	71 (44.5)	0.384
HDL (mg/dL)	53.5 (14.25)	57 (16)	0.220
Mature cEPCs (CD34^+^KDR^+^) (counts)	16 (18.25)	13 (16)	0.166
Immature cEPCs (CD34^+^KDR^+^CD133^+^) (counts)	12.5 (16.5)	10.5 (14.25)	0.130

Bold italicised values represent the p-values less than 0.05.

*BW* body weight, *BMI* body mass index, *HipC* hip circumference, *WC* waist circumference, *SBP* systolic blood pressure, *DBP* diastolic blood pressure, *ALT* alanine aminotransferase, *AST* aspartate aminotransferase, *CREAT* creatinine, *UA* uric acid, *FBS* fasting blood sugar, *CHOL* cholesterol, *TG* triglyceride, *HDL* high density lipoprotein cholesterol, *cEPC* circulating endothelial progenitor cells. Continuous variables were presented as their medians and interquartile ranges (IQR).

### The relationship between cEPC counts and depression associated clinical measurements

Table 2 shows the results of univariate and multiple linear regression for the association of cEPC counts with these clinical outcomes. In univariate analysis, SBP and DBP were significantly associated with immature (*p* = 0.001) and mature cEPC (*p* < 0.001) counts. In patients with MDD, the scores of HAMD, DSSS and DSSS-DS were significantly associated immature and mature cEPC levels (all p < 0.05), while the score of HAMA was significantly associated with the counts of immature cEPC (*p* = 0.015). The counts of immature and mature cEPC were also significantly associated with objective cognitive dysfunction measured by DSST (*p* = 0.002 for both immature and mature cEPC counts) and subjective cognitive dysfunction measured by PDQ-D (immature, *p* = 0.015; mature, *p* = 0.011). Regarding the participants’ generic health outcomes, both immature and mature cEPC counts were associated their perspective to mental health on SF12-MCS (all *p* = 0.006). Meanwhile, the counts of immature and mature cEPC were also significantly associated with the participants’ perspective to their social disability level as measured by SDS (*p* = 0.01 and 0.028 respectively). However, only the association of DSST with immature and mature cEPC counts remained statistically significant after correction for multiple comparisons. In multivariate stepwise linear regression, SBP was entered as a covariate. After controlling the confounding effect of SBP, only the HAMA scores were associated with immature (*β* = 1.35 (95% C.I. = 0.39–2.31), *p* = 0.007) and mature (*β* = 1.32 (95% C.I. = 0.12–2.57), *p* = 0.033) cEPC counts.

**Table 2:** Linear regression for the association of cEPC counts with clinical assessments related to major depressive disorder.

	Immature (CD34^+^KDR^+^CD133^+^)				Mature (CD34^+^KDR^+^)			
	Univariate		Multivariate		Univariate		Multivariate	
	β (95% C.I.)	p-value	β (95% C.I.)	p-value	β (95% C.I.)	p-value	β (95% C.I.)	p-value
SBP	0.35 (0.14, 0.55)	***0.001***	0.55 (0.24, 0.86)	***0.001***	0.48 (0.24, 0.72)	**< *0.001***	0.77 (0.37, 1.17)	**< *0.001***
DBP	0.40 (0.12, 0.67)	***0.006***			0.55 (0.22, 0.87)	***0.001***		
HAMD	1.09 (0.25, 1.93)	***0.012***			1.30 (0.28, 2.32)	***0.014***		
MADRS	0.57 (− 0.05, 1.18)	0.072			0.70 (− 0.05, 1.45)	0.067		
HAMA	1.18 (0.24, 2.12)	***0.015***	1.35 (0.39, 2.31)	***0.007***	1.14 (− 0.03, 2.31)	0.057	1.35 (0.12, 2.57)	***0.033***
DSST	− 0.32 (− 0.53, − 0.12)	***0.002***			− 0.41 (− 0.66, − 0.15)	***0.002***		
PDQ-D	0.74 (0.15, 1.32)	***0.015***			0.92 (0.22, 1.62)	***0.011***		
DSSS (total)	0.27 (0.07, 0.48)	***0.009***			0.31 (0.07, 0.56)	***0.012***		
DSSS-DS	0.50 (0.17, 0.84)	***0.003***			0.60 (0.20, 0.99)	***0.004***		
DSSS-SS	0.44 (− 0.03, 0.90)	0.064			0.48 (− 0.07, 1.03)	0.089		
DSSS-PS	0.43 (− 0.43, 1.30)	0.323			0.47 (− 0.57, 1.50)	0.373		
SF12-PCS	− 0.08 (− 0.55, 0.39)	0.737			0.01 (− 0.55, 0.57)	0.968		
SF12-MCS	− 0.31 (− 0.53, − 0.09)	***0.006***			− 0.37 (− 0.63, − 0.11)	***0.006***		
SDS (work/school)	0.99 (− 0.03, 2.01)	0.057			1.00 (− 0.23, 2.22)	0.109		
SDS (social)	1.33 (0.32, 2.35)	***0.010***			1.38 (0.16, 2.60)	***0.028***		
SDS (family life)	1.04 (− 0.05, 2.12)	0.061			1.05 (− 0.25, 2.35)	0.113		
SDS (total)	2.90 (0.54, 5.25)	0.016			3.30 (0.48, 6.11)	0.022		
GAF	− 0.50 (− 1.13, 0.13)	0.116			− 0.56 (− 1.33, 0.21)	0.153		

Bold italicised values represent the p-values less than 0.05.

*cEPCs* circulating endothelial progenitor cells, *SBP* systolic blood pressure, *DBP* diastolic blood pressure, *HAMD* Hamilton Depression Rating Scale, *MADRS* Montgomery–Åsberg Depression Rating Scale, *DSST* Digit Symbol Substitution Test, *HAMA* Hamilton Anxiety Rating Scale, *PDQ-D* Perceived Deficits Questionnaire-Depression, *DSSS* Depression and Somatic Symptom Scale, *DSSS-DS* DSSS-Depression Subscale, *DSSS-SS* DSSS-Somatic Subscale, *DSSS-PS* DSSS-Pain Subscale, *SF12-PCS* Physical Component Summary of the 12-Item Short Form Health Survey, *SF12-MCS* Mental Component Summary of the 12-Item Short Form Health Survey, *SDS* Sheehan Disability Scale, *GAF* Global Assessment of Functioning Scale.

## Discussion

In this study, we explored the role of cEPCs in MDD and MDD-related clinical presentations. Several studies have reported that MDD is associated with a decreased count or impaired function of cEPCs. For instance, the counts of cEPCs or their colony-forming units at diagnosis were significantly lower in patients with MDD than in controls^37,41,46^. The association of decreased cEPC count with MDD was also observed in patients with acute coronary syndrome comorbid with major depressive episode or mood and anxiety disorder^35,38^. However, in the present study, the counts of both types of cEPC in patients with MDD were not significantly different from that in HCs (Table 1). The discrepancy of our finding from others might stem from the effect of antidepressants on cEPC counts during depression treatment. In this study, we recruited patients with MDD who had an average illness duration of 4.4 ± 5.8 years and had been treated for an average of 0.8 ± 1.7 years. Antidepressants upregulate the protein expression of HIF1-alpha/VEGF cascades^47,48^, which then stimulates and promotes endothelial progenitor cell (EPC) mobilisation and proliferation and inhibits EPC apoptosis^49–51^, thus rescuing psychological stress-induced neovascularisation impairment^48^. Although Dome et al. reported that 1-month treatment during recovery from MDD could not alter peripheral EPC counts^36^, Lopez-Vilchez et al. found that a longer antidepressant treatment duration tended to increase cEPC counts^40^. Taken together, the data indicate that cEPC counts in our study’s patients with MDD possibly increased because of ongoing antidepressant treatment.

The relationships between depressive and anxiety severity and cEPC counts were also investigated. We observed a significant association between increased immature and mature cEPC counts and high HAMA scores, and also found trends of associations for the numbers of both type of cEPC and higher HAMD and DSSS-DS scores. These results indicate that increased immature and mature cEPC counts were associated with more severe anxiety level, and potentially with higher subjective and objective depressive symptom severity (assessed with DSSS-DS and HAMD respectively). Findings from several studies have shown that cEPC counts are inversely related to severity of depressive symptoms or stress. For instance, lower mature cEPC counts were associated with higher Depression Anxiety Stress Scale scores in healthy participants^33,34^, and immature cEPC counts negatively correlated with depression or anxiety severity in patients with acute coronary syndrome^38^. cEPC counts were lower in mothers of children with autism spectrum disorder than in those of neurotypical children^32^. Our results were contrary to these previous findings. Nevertheless, Riddell et al. demonstrated that progenitor cells are mobilised by acute psychological stress during a speech task and induced significant increases in the cEPC count^52^. Al et al. found that young individuals with cardiovascular risk factors express higher counts of circulating stem cells (CSCs; the parent cells of EPCs) than age-matched healthy individuals, but a similar risk factor load in older individuals is associated with lower counts of CSCs^53^. Similarly, higher CSC counts have been found to be associated with worsening insulin resistance in overweight and obese individuals, but reduced CSC counts develop early individuals with a history of type 2 diabetes mellitus and decline considerably in those with long-standing complicated diabetes mellitus^18,54,55^. These findings indicate compensatory release of CSCs by bone marrow in response to pathological stimuli or in the early stage of diseases. However, the marrow might be exhausted and hence decline to release CSCs in the late stage of diseases, possibly resulting in CSC counts being initially elevated but progressively reduced throughout the illness^18^. Therefore, our observed positive correlation of cEPCs with depressive severity might be a presentation of either an adaptive process or a compensatory attempt in the cEPC sources (such as bone marrow) in response to stress during a depressive episode. However, additional studies on patients with different stages of MDD are required to validate our findings.

Cognitive complaints, core symptoms of MDD, can be identified at MDD onset, often persist in euthymic phases and have been suggested as critical mediators of adverse psychosocial outcomes in patients with MDD^42^. A determining factor contributing to cognitive deficit is chronic cerebral hypoperfusion, in which the supply of oxygen, glucose, and other essential nutrients might not be constant and adequate^56,57^. Studies have indicated cognitive deficit levels in MDD are associated with cerebral microvascular dysfunction^58,59^. Cerebral microvascular dysfunction (or small vessel diseases) is considered a manifestation of endothelial dysfunction that can be attenuated by cEPCs^60^ because the cell populations are involved in endothelial and neurovascular repair, blood–brain barrier establishment, and cerebral microvascular remodelling^61^. In addition, available evidence indicates cerebral blood flow is correlated with cEPC level^62,63^. Given the convergent evidence linking cognitive deficit, cerebral microvascular dysfunction and effect of cEPCs on cerebral perfusion, we further analysed the relationship between the cEPC count and cognitive deficit in MDD. We observed that the level of objective (DSST) and subjective (PDQ-D) cognitive deficit were potentially associated with increased counts of immature and mature cEPCs in the univariate analysis (Table 2), suggesting that individuals with higher cEPC counts exhibited worse cognitive performance and greater cognitive deficit. Studies have investigated the relationship of cEPC counts with cognitive function in healthy individuals or in individuals with dementia. In general, low cEPC counts were correlated with greater yearly cognitive decline, worse immediate verbal and visual memory, and worse delayed verbal memory in healthy individuals^63,64^ or in patients with Alzheimer’s disease^65,66^. The correlation direction of cEPC counts and cognitive performance indicated in these studies is contrary to our findings. The use of different populations across different studies makes interpretation of these results challenging. By contrast, the cEPC count has been found to increase in the early or acute phase of cerebrovascular injury, such as ischaemic stroke or intracerebral haemorrhage^29,30^, suggesting there is a cellular compensatory attempt or behaviour of cEPCs in response to cerebrovascular injury. Increased cEPC counts have also been associated with decreased infarct growth and intracerebral haemorrhage volume and positive neurological and functional outcomes^29–31^. If cognitive dysfunction is considered a clinical surrogate of cerebral microvascular and endothelial dysfunction^58–60^, the associations of increased cEPC counts with more severe cognitive deficit and subjective impairment in our study might also be a cellular adaptive process to subtle or silent cerebral microvascular dysfunction.

Compared with previous studies that only used simple depression questionnaires, our study contains more comprehensive measurements of MDD, including self-assessed life quality, functioning, and social disability. One of our key findings in the univariate analysis was the potential association of increased immature and mature cEPC counts with worse mental life quality (SF-MCS) and poor social disability in SDS (Table 2). The release from bone marrow stroma, mobilisation, and vasculogenesis functions of cEPCs depends on matrix metalloproteinase-9 (MMP-9) activation^20,67,68^. Yoshida et al. showed that quality of life in patients with MDD was negatively correlated with the concentration of MMP-9^69^. Although no other studies have investigated the relationship of cEPCs with MDD-related psychosocial outcomes, the combined evidence from Yoshida et al. and our finding might indicate that regulation of cEPC functions is related to many aspects of MDD—not only mood, anxiety, and cognitive symptoms but also psychosocial outcome.

In the present study, we found patients with MDD had significantly higher SBP and DBP than the HCs (Table 1). In a meta-analysis summarizing longitudinal studies among initially somatic-disease free subjects, depression increases the risk of subsequent hypertension^70^. Although our study is cross-sectional, our finding supports the association of depression and elevated blood pressures. There have been several proposed mechanisms linking depression, elevated blood pressures and risk of cardiovascular diseases, such like unhealthy lifestyle, genetic pleiotropy, dysregulations in immono-inflammatory, autonomic and hypothalamic–pituitary–adrenal systems and antidepressant use^71^. These factors may interplay in vicious cycle through which depression and cardiovascular disease impact on each other^71^.

The present study has some limitations. First, our study is a cross-sectional study with a small sample size, precluding causal interpretations for the association of cEPC levels with mood severities, cognitive performance, life quality and functional outcomes. Additional longitudinal studies with larger samples are needed to explore the temporal relationship of cEPCs and antidepressant treatment throughout the course of MDD. In addition, cEPC counts can be affected by confounding factors such as physical activities, exercise, and other oxidative stress biomarkers. The proximate cause should be carefully identified. Third, only the association of anxiety levels and increased cEPC counts was robust after correcting the confounding effect of SBP in multivariate linear regression. The associations of cEPC counts with the level of depressed mood, cognitive deficit, subjective mental life quality and subjective social disability in univariate analysis were not significant after multiple comparison adjustments. However, we think our study might be an exploratory study in which the data were collected from clinical observation and assessments and tested for descriptive purpose but not for confirmatory purpose or decision making^72^. Imposing a strict cut-off on statistical significance with forced adjustment of multiple comparisons in exploratory studies might lose some novel information for further hypothesis generation. Therefore, some researchers have suggested that the adjustment for multiple comparisons in exploratory analyses might not be necessary^72–74^. Despite these shortcomings, our study is to the best of our knowledge the most comprehensive one investigating the relationship between MDD and cEPC counts from different clinical perspectives because we assessed not only subjective and objective mood severity but also cognitive dysfunction and functional disability for association with cEPCs. Subsequent study should be conducted to confirm these observed associations.

## Materials and methods

### Participants

The study was conducted in the psychiatric outpatient department of Taipei Veterans General Hospital (Taipei, Taiwan) including patients aged 20–65 years who met the *Diagnostic and Statistical Manual of Mental Disorders, Fifth Edition* (DSM-5) criteria for MDD with a Clinical Global Impression-Severity (CGI-S) scale score for depression of ≤ 3 were included. The Clinical Global Impression-Severity Scale is a 7-point observer-rated scale that requires the clinician to measure the illness severity for the patients with mental disorders, relative to the clinician’s past experience with patients with the same diagnosis^75^. The score equal or less than 3 means the patients in the study were normal to mildly ill at recruitment and able to complete other assessments^75^. Age and gender-matched participants without any psychiatric illness were enrolled as the healthy control (HC) group. The exclusion criteria included any DSM-5 diagnosis of the following: lifetime history of schizophrenia or any other psychosis, intellectual disability, organic mental disorder, autoimmune or immune diseases, substance abuse in the past 3 months or substance dependence in the past 6 months, pregnancy or breastfeeding, and unstable physical illnesses.

This study was approved by the Institutional Review Board of the Taipei Veterans General Hospital and conducted in accordance with the Declaration of Helsinki. Written informed consent was obtained from all patients prior to their study inclusion.

### Clinical assessments

Clinical symptoms were assessed by a psychiatrist by using the 17-item Hamilton Depression Rating Scale, (HAMD), Montgomery–Åsberg Depression Rating Scale (MADRS), and Hamilton Anxiety Rating Scale (HAMA), respectively. HAMD and MADRS are two popular scales among a number of self-report and clinician ratings for depressive symptom severity. However, neither the HAMD nor the MADRS evaluate all of the core criterion symptoms of a major depressive episode. The HAMD lacks ratings of oversleeping, overeating and concentration, while the MADRS lacks ratings of interest, guilt and psychomotor changes^76^. In that regard, both ratings were used to fully assess depressive symptoms severity in the MDD patients.

The participants’ objective neurocognitive functions were also assessed with the Digit Symbol Substitution Test (DSST), which evaluates psychomotor speed of performance requiring visual perception, spatial decision-making, and motor skills; it is a valid and sensitive measure of various cognitive domains including executive function, attention, psychomotor speed, and working memory^77^. In addition, the participants self-assessed their subjective cognitive deficits with the Perceived Deficits Questionnaire-Depression (PDQ-D)^78^, which measures the impact of cognitive dysfunction on participants’ daily life on the basis of their own experience and perceptions when having depressive symptoms.

The participants also completed another two self-administered questionnaires: the Depressive and Somatic Symptom Scale (DSSS) and the 12-Item Short Form Health Survey (SF-12). The DSSS is composed of a depression subscale (DSSS-DS), somatic subscale (DSSS-SS), and pain subscale (DSSS-PS)^79^. The DSSS has the advantage of simultaneously assessing both somatic and depression symptoms, overcoming the deficiencies of other depression scales with few somatic items. The SF-12, a 12-item questionnaire used to assess generic health-related quality of life from the patient’s perspective, comprises a physical component summary (SF12-PCS) and mental component summary (SF12-MCS)^80^.

Finally, the individual’s functional impairment in school/work, social, or family life and overall functioning in daily life were evaluated with the Sheehan Disability Scale (SDS) and Global Assessment of Functioning Scale (GAF), respectively. SDS is a participant-rated tool evaluating functional disability in work, school, social, and family life with only three self-rated items^81^, whereas GAF is a clinical assessment of an individual’s overall functioning levels comprising social, occupational, and psychological functioning during a given period based on a psychiatrist’s judgement.

### Measurements for biochemical parameters and cEPC counts

Blood samples were drawn after a 12-h overnight fasting. Plasma biochemical parameters, such as alanine aminotransferase (ALT), aspartate aminotransferase (AST), creatinine (CREAT), fasting glucose (FBS), triglyceride (TG), cholesterol (CHOL), high-density lipoprotein cholesterol (HDL-C) and uric acid (UA) levels, were determined using standard laboratory procedures.

The cEPC count was measured through flow cytometry conducted by researchers who were blinded to all clinical data. A 1.0-mL sample of peripheral blood was obtained from each participant. Blood samples were subsequently incubated with allophycocyanin (APC)-conjugated monoclonal antibodies against human KDR (R&D, Minneapolis, MN, USA), phycoerythrin (PE)-conjugated monoclonal antibodies against human CD133 (Miltenyi Biotec, Germany), and fluorescein isothiocyanate (FITC)-conjugated monoclonal antibodies against human CD34 (BD Biosciences Pharmingen, San Diego, CA, USA) in the dark for 30 min. Each analysis was based on 150,000 acquired events, which was as reliable as 500,000 events (intraclass correlation coefficient > 0.95). When viability markers were included, the viability of the cEPCs was up to 96.3%. The interindividual variability of two separate samples obtained from 10 patients was strongly correlated (*r* = 0.90, *p* < 0.001). The intraindividual variability of immature (CD34^+^KDR^+^CD133^+^) and mature (CD34^+^KDR^+^) cEPCs over time was evaluated in 21 patients through two measurements 1 year apart, and the resulting intraclass correlation coefficients were 0.69, 0.75, and 0.78 respectively. Cell counts are expressed as cEPCs per 10^5^ mononuclear cells.

### Statistical analyses

Statistical analysis was performed using SPSS (version 21; SPSS Inc., Chicago, IL, USA). The difference in the distributions of categorical variables between groups were compared using the chi-square test (and Fisher’s exact test if necessary). To compare continuous variables between groups, two-tailed independent *t* tests were used if the outcomes were approximately normally distributed, or Mann–Whitney *U* tests if the outcomes were deviated from normal distribution. The distributions of continuous variables were examined with Shapiro–Wilk tests. Meanwhile, linear regressions were performed to adjust for potential confounding effects on continuous outcomes since linear regression is an appropriate analysis when the dependent variable is not normally distributed^82^. In univariate linear analysis, the threshold of statistical significance was set at *p* < 0.0028 (0.05/18) to control the inflation of type I error. For multivariate stepwise linear analysis, the threshold of statistical significance was set at *p* < 0.05.

## References

[1] GS Collaborators (2017). Measuring progress and projecting attainment on the basis of past trends of the health-related Sustainable Development Goals in 188 countries: An analysis from the Global Burden of Disease Study 2016. Lancet.

[2] Mathers CD, Loncar D (2006). Projections of global mortality and burden of disease from 2002 to 2030. PLoS Med..

[3] Dhar AK, Barton DA (2016). Depression and the link with cardiovascular disease. Front. Psychiatry.

[4] Pratt LA, Ford DE, Crum RM, Armenian HK, Gallo JJ, Eaton WW (1996). Depression, psychotropic medication, and risk of myocardial infarction. Prospective data from the Baltimore ECA follow-up. Circulation.

[5] Lippi G, Montagnana M, Favaloro EJ, Franchini M (2009). Mental depression and cardiovascular disease: A multifaceted, bidirectional association. Semin. Thromb. Hemost..

[6] Chaddha A, Robinson EA, Kline-Rogers E, Alexandris-Souphis T, Rubenfire M (2016). Mental health and cardiovascular disease. Am. J. Med..

[7] Huffman JC, Celano CM, Beach SR, Motiwala SR, Januzzi JL (2013). Depression and cardiac disease: Epidemiology, mechanisms, and diagnosis. Cardiovasc. Psychiatry Neurol..

[8] Davidson KW, Korin MR (2010). Depression and cardiovascular disease: Selected findings, controversies, and clinical implications from 2009. Clevel. Clin. J. Med..

[9] Kahl KG, Stapel B, Frieling H (2019). Link between depression and cardiovascular diseases due to epigenomics and proteomics: Focus on energy metabolism. Prog. Neuropsychopharmacol. Biol. Psychiatry.

[10] Antonogeorgos G (2012). Understanding the role of depression and anxiety on cardiovascular disease risk, using structural equation modeling; the mediating effect of the Mediterranean diet and physical activity: The ATTICA study. Ann. Epidemiol..

[11] Georgousopoulou EN (2014). Association between mediterranean diet and non-fatal cardiovascular events, in the context of anxiety and depression disorders: A case/case–control study. Hellenic J. Cardiol..

[12] Mamplekou E (2010). Urban environment, physical inactivity and unhealthy dietary habits correlate to depression among elderly living in eastern Mediterranean islands: The MEDIS (MEDiterranean ISlands Elderly) study. J. Nutr. Health Aging.

[13] De Hert M, Detraux J, Vancampfort D (2018). The intriguing relationship between coronary heart disease and mental disorders. Dialogues Clin. Neurosci..

[14] Peters A, McEwen BS (2015). Stress habituation, body shape and cardiovascular mortality. Neurosci. Biobehav. Rev..

[15] Halaris A (2013). Inflammation, heart disease, and depression. Curr. Psychiatry Rep..

[16] Halaris A (2017). Inflammation-associated co-morbidity between depression and cardiovascular disease. Curr. Top. Behav. Neurosci..

[17] Mechawar N, Savitz J (2016). Neuropathology of mood disorders: Do we see the stigmata of inflammation?. Transl. Psychiatry.

[18] Fadini GP (2019). Circulating stem cells and cardiovascular outcomes: From basic science to the clinic. Eur. Heart J..

[19] Asahara T (1997). Isolation of putative progenitor endothelial cells for angiogenesis. Science.

[20] Hristov M, Erl W, Weber PC (2003). Endothelial progenitor cells: Mobilization, differentiation, and homing. Arterioscler. Thromb. Vasc. Biol..

[21] Friedrich EB, Walenta K, Scharlau J, Nickenig G, Werner N (2006). CD34^−^/CD133^+^/VEGFR-2^+^ endothelial progenitor cell subpopulation with potent vasoregenerative capacities. Circ. Res..

[22] Pinho S, Frenette PS (2019). Haematopoietic stem cell activity and interactions with the niche. Nat. Rev. Mol. Cell Biol..

[23] Rabelink TJ, de Boer HC, de Koning EJ, van Zonneveld AJ (2004). Endothelial progenitor cells: More than an inflammatory response?. Arterioscler. Thromb. Vasc. Biol..

[24] Simard T (2017). Progenitor cells for arterial repair: Incremental advancements towards therapeutic reality. Stem Cells Int..

[25] Fadini GP (2006). Peripheral blood CD34^+^KDR^+^ endothelial progenitor cells are determinants of subclinical atherosclerosis in a middle-aged general population. Stroke.

[26] Hill JM (2003). Circulating endothelial progenitor cells, vascular function, and cardiovascular risk. N. Engl. J. Med..

[27] Greden JF (2003). Physical symptoms of depression: Unmet needs. J. Clin. Psychiatry.

[28] Luo S, Xia W, Chen C, Robinson EA, Tao J (2016). Endothelial progenitor cells and hypertension: Current concepts and future implications. Clin. Sci. (Lond.).

[29] Pias-Peleteiro J (2016). Increased endothelial progenitor cell levels are associated with good outcome in intracerebral hemorrhage. Sci. Rep..

[30] Yip HK (2008). Level and value of circulating endothelial progenitor cells in patients after acute ischemic stroke. Stroke.

[31] Sobrino T (2007). The increase of circulating endothelial progenitor cells after acute ischemic stroke is associated with good outcome. Stroke.

[32] Aschbacher K (2017). Chronic stress is associated with reduced circulating hematopoietic progenitor cell number: A maternal caregiving model. Brain Behav. Immun..

[33] Chen H, Yiu KH, Tse HF (2011). Relationships between vascular dysfunction, circulating endothelial progenitor cells, and psychological status in healthy subjects. Depress. Anxiety.

[34] Chen H (2013). Relationship of depression, stress and endothelial function in stable angina patients. Physiol. Behav..

[35] Di Stefano R (2014). Impact of depression on circulating endothelial progenitor cells in patients with acute coronary syndromes: A pilot study. J. Cardiovasc. Med. (Hagerstown).

[36] Dome P (2012). Investigation of circulating endothelial progenitor cells and angiogenic and inflammatory cytokines during recovery from an episode of major depression. J. Affect. Disord..

[37] Dome P (2009). Circulating endothelial progenitor cells and depression: A possible novel link between heart and soul. Mol. Psychiatry.

[38] Felice F (2015). Influence of depression and anxiety on circulating endothelial progenitor cells in patients with acute coronary syndromes. Hum. Psychopharmacol..

[39] Fiedorowicz JG, Ellingrod VL, Kaplan MJ, Sen S (2015). The development of depressive symptoms during medical internship stress predicts worsening vascular function. J. Psychosom. Res..

[40] Lopez-Vilchez I (2016). Endothelial damage in major depression patients is modulated by SSRI treatment, as demonstrated by circulating biomarkers and an in vitro cell model. Transl. Psychiatry.

[41] Yang L (2011). Depression is associated with lower circulating endothelial progenitor cells and increased inflammatory markers. Acta Neuropsychiatr..

[42] Chakrabarty T, Hadjipavlou G, Lam RW (2016). Cognitive dysfunction in major depressive disorder: Assessment, impact, and management. Focus (Am. Psychiatr. Publ.).

[43] Fu X, Zhang F, Liu F, Yan C, Guo W (2019). Editorial: Brain and somatization symptoms in psychiatric disorders. Front. Psychiatry.

[44] Simon GE, VonKorff M (1991). Somatization and psychiatric disorder in the NIMH Epidemiologic Catchment Area study. Am. J. Psychiatry.

[45] Gupta S, Goren A, Dong P, Liu D (2016). Prevalence, awareness, and burden of major depressive disorder in urban China. Expert Rev. Pharmacoecon. Outcomes Res..

[46] Blum A, Pastukh N, Zaroura I, Rotem J, Kamal F (2017). Impaired ability to grow colonies of endothelial stem cells could be the mechanism explaining the high cardiovascular morbidity and mortality of patients with depression. QJM.

[47] Hu Q (2020). Effect of fluoxetine on HIF-1alpha-Netrin/VEGF cascade, angiogenesis and neuroprotection in a rat model of transient middle cerebral artery occlusion. Exp. Neurol..

[48] Maingrette F (2015). Psychological stress impairs ischemia-induced neovascularization: Protective effect of fluoxetine. Atherosclerosis.

[49] Liu Y (2018). Knockdown of HIF-1alpha impairs post-ischemic vascular reconstruction in the brain via deficient homing and sprouting bmEPCs. Brain Pathol..

[50] Zan T, Li H, Du Z, Gu B, Liu K, Li Q (2015). Enhanced endothelial progenitor cell mobilization and function through direct manipulation of hypoxia inducible factor-1alpha. Cell Biochem. Funct..

[51] Milkiewicz M, Ispanovic E, Doyle JL, Haas TL (2006). Regulators of angiogenesis and strategies for their therapeutic manipulation. Int. J. Biochem. Cell Biol..

[52] Riddell NE (2015). Progenitor cells are mobilized by acute psychological stress but not beta-adrenergic receptor agonist infusion. Brain Behav. Immun..

[53] Al Mheid I (2016). Age and human regenerative capacity impact of cardiovascular risk factors. Circ. Res..

[54] Fadini GP (2015). Circulating stem cells associate with adiposity and future metabolic deterioration in healthy subjects. J. Clin. Endocrinol. Metab..

[55] Fadini GP (2010). Time course and mechanisms of circulating progenitor cell reduction in the natural history of type 2 diabetes. Diabetes Care.

[56] De Silva TM, Faraci FM (2016). Microvascular dysfunction and cognitive impairment. Cell. Mol. Neurobiol..

[57] Hamel E, Nicolakakis N, Aboulkassim T, Ongali B, Tong XK (2008). Oxidative stress and cerebrovascular dysfunction in mouse models of Alzheimer's disease. Exp. Physiol..

[58] Smith PJ, Blumenthal JA, Hinderliter AL, Watkins LL, Hoffman BM, Sherwood A (2018). Microvascular endothelial function and neurocognition among adults with major depressive disorder. Am. J. Geriatr. Psychiatry.

[59] van Agtmaal MJM, Houben A, Pouwer F, Stehouwer CDA, Schram MT (2017). Association of microvascular dysfunction with late-life depression: A systematic review and meta-analysis. JAMA Psychiat..

[60] Maki T (2018). Endothelial progenitor cell secretome and oligovascular repair in a mouse model of prolonged cerebral hypoperfusion. Stroke.

[61] Malinovskaya NA (2016). Endothelial progenitor cells physiology and metabolic plasticity in brain angiogenesis and blood–brain barrier modeling. Front. Physiol..

[62] Taguchi A (2004). Circulating CD34-positive cells provide an index of cerebrovascular function. Circulation.

[63] Nation DA (2018). Circulating progenitor cells correlate with memory, posterior cortical thickness, and hippocampal perfusion. J. Alzheimers Dis..

[64] Hajjar I, Goldstein FC, Waller EK, Moss LD, Quyyumi A (2016). Circulating progenitor cells is linked to cognitive decline in healthy adults. Am. J. Med. Sci..

[65] Kong XD, Zhang Y, Liu L, Sun N, Zhang MY, Zhang JN (2011). Endothelial progenitor cells with Alzheimer's disease. Chin. Med. J. (Engl.).

[66] Lee ST (2009). Reduced circulating angiogenic cells in Alzheimer disease. Neurology.

[67] Huang PH (2009). Matrix metalloproteinase-9 is essential for ischemia-induced neovascularization by modulating bone marrow-derived endothelial progenitor cells. Arterioscler. Thromb. Vasc. Biol..

[68] Heissig B (2002). Recruitment of stem and progenitor cells from the bone marrow niche requires MMP-9 mediated release of kit-ligand. Cell.

[69] Yoshida T (2012). Decreased serum levels of mature brain-derived neurotrophic factor (BDNF), but not its precursor proBDNF, in patients with major depressive disorder. PLoS One.

[70] Meng L, Chen D, Yang Y, Zheng Y, Hui R (2012). Depression increases the risk of hypertension incidence: A meta-analysis of prospective cohort studies. J. Hypertens..

[71] Penninx BW (2017). Depression and cardiovascular disease: Epidemiological evidence on their linking mechanisms. Neurosci. Biobehav. Rev..

[72] Bender R, Lange S (2001). Adjusting for multiple testing—When and how?. J. Clin. Epidemiol..

[73] Althouse AD (2016). Adjust for multiple comparisons? It's not that simple. Ann. Thorac. Surg..

[74] Rothman KJ (1990). No adjustments are needed for multiple comparisons. Epidemiology.

[75] Guy W (1976). Assessment Manual for Psychopharmacology.

[76] Carmody TJ (2006). The Montgomery Asberg and the Hamilton ratings of depression: A comparison of measures. Eur. Neuropsychopharmacol..

[77] Jaeger J (2018). Digit symbol substitution test: The case for sensitivity over specificity in neuropsychological testing. J. Clin. Psychopharmacol..

[78] Lam RW (2018). Psychometric validation of the Perceived Deficits Questionnaire-Depression (PDQ-D) instrument in US and UK respondents with major depressive disorder. Neuropsychiatr. Dis. Treat..

[79] Hung CI, Liu CY, Juang YY, Wang SJ (2006). The impact of migraine on patients with major depressive disorder. Headache.

[80] Ware J, Kosinski M, Keller SD (1996). A 12-Item Short-Form Health Survey: Construction of scales and preliminary tests of reliability and validity. Med. Care.

[81] Sheehan KH, Sheehan DV (2008). Assessing treatment effects in clinical trials with the discan metric of the Sheehan Disability Scale. Int. Clin. Psychopharmacol..

[82] Li X, Wong W, Lamoureux EL, Wong TY (2012). Are linear regression techniques appropriate for analysis when the dependent (outcome) variable is not normally distributed?. Investig. Ophthalmol. Vis. Sci..

